# Molluscum contagiosum survey – common approach and attitude towards treatment and research in Dutch general practice

**DOI:** 10.1186/s12875-023-02226-y

**Published:** 2023-12-07

**Authors:** Roeland M. Watjer, Tobias N. Bonten, Koen D. Quint, Mohammad M. Hasani, Mattijs E. Numans, Just A.H. Eekhof

**Affiliations:** 1 Department of Public Health and Primary Care, Leiden University Medical Center (LUMC), Albinusdreef 2, Leiden, 2333 ZA The Netherlands; 2 Department of Dermatology, Leiden University Medical Center (LUMC), Albinusdreef 2, 2333 ZA Leiden, The Netherlands; 3 Department of Dermatology, Roosevelt Clinic, Rooseveltstraat 82A, 2321 BM Leiden, The Netherlands

**Keywords:** Molluscum contagiosum, Treatment, Survey, General practice

## Abstract

**Background:**

Molluscum contagiosum (MC) can cause significant burden in children. So far, pharmacological treatment has not been proven beneficial. More rigorous interventions have not been well studied. Current guidelines advise a “wait and see” policy. However, children and their parents frequently visit their GP requesting intervention. Therefore, the aim of this study was to gain insight into the approach to MC by GPs and parents’ expectations and to investigate willingness to participate in an interventional study.

**Methods:**

A survey study was carried out among GPs and parents using a questionnaire for each group inquiring about MC and potential study participation. Descriptive statistics were used to analyze results and logistical regression to investigate factors influencing participation.

**Results:**

The majority of GPs (88%) preferred an expectative approach; only 21% were willing to participate in a trial as proposed. GPs estimating ≥ 50% of parents would request treatment, were more likely to participate. Most responding parents did or would visit their GP requesting treatment. In contrast to GPs, 58% were willing to participate. Parents preferring cryotherapy or curettage were more likely to participate.

**Conclusion:**

Our study demonstrated that the majority of GPs preferred a conservative approach, adhering to current guidelines. However, most parents preferred treatment to resolve MC and symptoms. Parents’ willingness to participate was much higher than GP’s, reflecting parents’ desire for treatment. These findings underscore the need for continued therapeutic research. Careful preparation and selection of GPs and patients will be essential to ensure the feasibility of such an endeavor.

**Trial registration:**

This survey study was not part of a clinical trial.

**Supplementary Information:**

The online version contains supplementary material available at 10.1186/s12875-023-02226-y.

## Background

Molluscum contagiosum (MC), also called ‘water warts’ referring to the dome-shaped papules mimicking water droplets, is caused by the molluscum contagiosum virus (MCV). MCV is the only virus of the molluscipoxvirus genus and part of the chordopoxvirinae subfamily of poxviridiae [1]. This virus spreads by close skin-to-skin contact primarily affecting young children [2, 3]. Infection in childhood normally provokes a strong immune response, creating long-lasting immunity and making MC more common in children than in adults [4, 5]. However, MC can also affect adults, especially immunocompromised individuals [2, 6].

MC is relatively common in Dutch general practice with a cumulative incidence of 17 per 1000 person-years in children [7]. Similar rates are found in the UK and North America [2, 8–10].

The dome-shaped, umbilicated papules are pathognomonic for MC. Mostly appearing on the trunk, antecubital fossae, popliteal fossae, groin, and axillae, MC lesions may present anywhere on the body except the palms and soles of the feet [11]. Although frequently considered a nuisance, MC lesions have a median time to resolution of 12 months (IQR 8–18) and can significantly impact children’s quality of life; [12] more lesions and longer duration are associated with a greater negative effect [12]. Especially in atopic children, symptoms can be substantial [12, 13].

Parents or caregivers (henceforth referred to as parents) consult their GP not only for diagnosis or concern for possible spread but also to request treatment, especially in case of numerous lesions or pronounced symptoms [14, 15]. If considered, cryotherapy and curettage are the preferred treatment options [16]. However, there is a lack of evidence for these destructive treatments and pharmacological treatments have not been proven beneficial, while potentially having side effects such as skin irritation or scar formation [17]. Consequently, current guidelines of the Dutch College of General Practitioners, American Academy of Dermatology, and National Institute for Health and Care Excellence (NICE), advise an expectative approach, i.e. wait-and-see [18–20]. However, effective destructive treatment could resolve symptoms more quickly, prevent the development of new lesions and decrease the spread to others [21, 22].

The Dutch College for General Practitioners has put the investigation of treatments for MC on its research agenda [23]. Therefore, a research proposal was drafted for a randomized trial comparing the effectiveness of cryotherapy or curettage (under local anesthesia with lidocaine/prilocaine ointment) to the currently advised conservative approach, i.e. watchful waiting, in a three-arm trial. Given the current guidelines and considering the feasibility of such a trial, one first would need to establish the usual approach to MC and willingness to participate.

We therefore conducted a survey on the current approach, experience, and preferences of GPs and parents regarding MC, and the extent to which both groups would be willing to participate in a randomized trial as proposed.

## Methods

### Study design

Two separate questionnaires were constructed, one for GPs and one for parents of children having, having had, or potentially acquiring MC. In both surveys in Dutch, a general introduction to the survey and a short background of MC were provided; the questions regarding the interventional study were introduced by describing the proposed trial, comparing usual care i.e. conservative treatment to either cryotherapy or curettage.

The questionnaire for GPs consisted of 18 substantive questions of which eight concerning the common/preferred approach and experience with MC treatment, seven about the willingness to participate, and three about the responding GPs’ general characteristics (age, gender, and working experience) (See Appendix 1).

The questionnaire for parents consisted of 15 substantive questions, of which eight questions were about their experience with MC, children’s symptoms, and possible reasons to consult their GP, four inquired about the willingness to let their children participate, and three about the responding parent’s and their children’s general characteristics (age, gender, number of children, children’s age) (See Appendix 2).

This survey study was approved by the designated medical ethics committee for non-WMO*^1^ studies of the Leiden University Medical Center (LUMC) on April 25th, 2022.

### Data collection

The survey request for GPs was distributed through social media and our regional primary care research collaboration of LUMC affiliated general practices, the Extramural LUMC Academic Network (ELAN). The survey request for parents was distributed through social media, at local schools and sports facilities.

Both questionnaires were administered online using Castor’s Electronic Data Capture (EDC), a cloud-based data management software program. Both survey requests contained the necessary link or QR code for the online survey. GPs and parents could only fill out the questionnaire anonymously.

### Respondents

Regarding GPs, no selection criteria were applied. Respondents to the survey for parents had to confirm being aged 18 years or over, and being an actual parent or caregiver. Otherwise, no additional criteria were required.

Participation was on a voluntary basis; no standard remuneration was offered. However, as an incentive five skin curettes and five bottles of spring water were raffled off amongst GPs; for parents, five €25 vouchers for an online store were raffled off amongst the respondents. The filled-out surveys were not linked to the participants’ email addresses used for the raffle, thus preserving the anonymity of the respondents’ answers given.

### Sample size

The sample size calculation was based on the question regarding the potential willingness to participate in an interventional study as proposed. Considering the chance of the respondent’s answer being equally distributed between yes or no, i.e. 50%, subsequently applying a confidence limit of 10% (above and below) and a confidence interval of 95%, the calculated sample size for the required respondents was n = 97 for both surveys.

### Statistical analysis

The answers of both responding GPs and parents were analyzed using descriptive statistics. Logistical regression analysis was performed to describe the association between GP characteristics, their common/preferred approach and experience with MC, and their willingness to participate in interventional research as proposed. In parallel, regarding the answers of parents, the association between parent’s characteristics (age, gender, number of children, children’s age) and experience with MC, and their willingness to participate in a study as proposed was also analyzed using logistical regression.

### Statistical software

Statistical analyses were conducted using IBM SPSS (version 25.0.0.0).

## Results

### Survey response

**Fig. 1:**
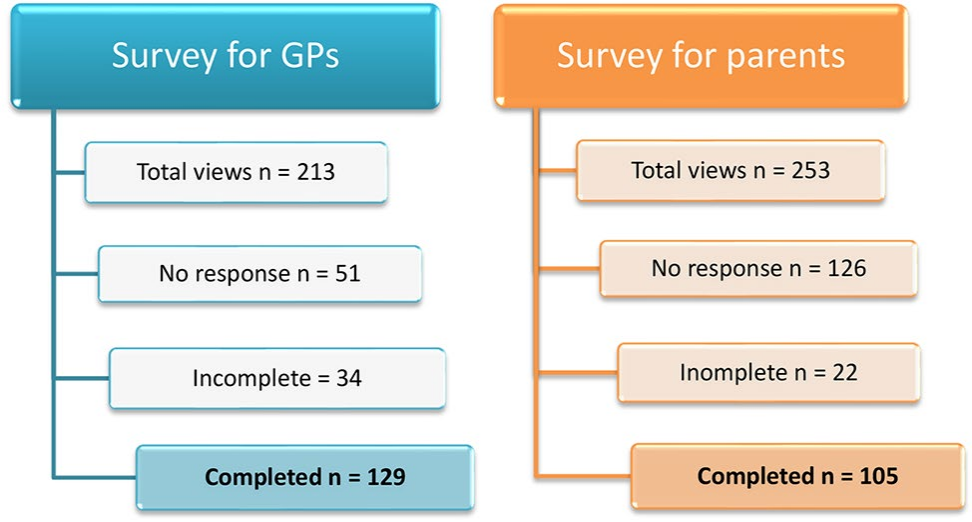
Flow-chart for survey response

The survey for GPs was available from May 18th until November 18th, 2022, reaching the required number of respondents. Equally, the survey for parents was open from June 20th until November 20th, 2022. Figure 1 illustrates how many times the survey was only opened i.e. views, how many provided no response at all, or did not complete the survey. A total of 129 GPs and 105 parents completed the survey, above the required number due to weekly checks of response rate. Only completed surveys were analyzed.

### Baseline characteristics

Table 1 shows the baseline characteristics of responding GPs and parents. The majority of GPs were female (73.6%), over 40 years old (65.9%), and with an average working experience of 13.7 years.

**Table 1:** Baseline characteristics of respondents

**GP characteristics (N = 129)**	
Mean age in years (SD, range)	45.1 (9.7, 27–69)
Aged ≥ 40 (%)	85 (65.9)
Aged ≥ 50 (%)	38 (29.5)
Gender, female (%)	95 (73.6)
Mean working experience in years (SD, range)	13.7 (9.4, 1–40)
**Parent and children characteristics (N = 105)**	
Parent’s age in years, mean (SD)	38.9 (5.4)
Parent’s gender, female (%)	92 (87.6)
Number of children, mean (SD)	2.3 (0.8)
Number of children per parent	
1 child (%)	15 (14.3)
2 children (%)	56 (53.3)
3 children (%)	27 (25.7)
4 children (%)	7 (6.7)
Children’s mean age in years (SD, range)	6.4 (3.9, 0–18)
Children’s mean age in years per child	
Mean age 1st child (SD, range)	8.0 (4.0, 0–18)
Mean age 2nd child (SD, range)	5.7 (3.7, 0–16)
Mean age 3rd child (SD, range)	3.9 (4.0, 0–15)
Mean age 4th child (SD, range)	3.1 (2.7, 0–7)

N = number of responding GPs/parents, SD = standard deviation

Responding parents were aged 38.9 years on average, the majority female (87.6%) with 2 children (53.3%). The children were aged 0 and 18, with a mean of 6.4 years.

### MC treatment experience, approach, and preferences

Table 2 represents an overview of GP’s general experience with MC, common approach, and treatment preferences. Regarding experience, most GPs estimated seeing 5–10 MC patients yearly, of which one-third would request treatment. Most did not have experience with cryotherapy or curettage for MC. The preferred approach was expectative, followed by an antipruritic, both in general and in case treatment was requested. Cryotherapy was considered more often in case of a treatment request.

**Table 2:** GP experience and treatment preferences

**Estimated number of MC patient visits (N = 129)**	
0–5/year (%)	34 (26.4)
5–10/year (%)	60 (46.5)
10–20/year (%)	28 (21.7)
20–50/year (%)	7 (5.4)
**Estimated % of parents requesting treatment for MC**	
Mean % (SD)	33.0 (26.9)
**Experience with cryotherapy for MC (N = 129)**	
Any experience (%)	42 (32.6)
No experience (%)	87 (67.4)
**Experience with curettage for MC (N = 129)**	
Any experience (%)	24 (18.6)
No experience (%)	105 (81.4)
**Currently applying cryotherapy or curettage (N = 129)**	
Cryotherapy (%)	25 (19.4)
Curettage (%)	13 (10.1)
Preferred treatment in general* (N = 129)	
Expectative (%)	113 (87.6)
Antipruritic (%)	58 (45.0)
Topical corticosteroid (%)	10 (7.8)
Curettage (%)	6 (4.7)
Cryotherapy (%)	4 (3.1)
Topical ointment against MC (%)	1 (0.8)
Other (%)	7 (5.4)
Preferred treatment in case of a treatment request* (N = 129)	
Expectative (%)	75 (58.1)
Antipruritic (%)	41 (31.8)
Cryotherapy (%)	30 (23.3)
Topical corticosteroid (%)	15 (11.6)
Curettage (%)	14 (10.9)
Topical ointment against MC (%)	6 (4.7)
Other (%)	4 (3.1)

MC = molluscum contagiosum, N = number of responding GPs, SD = standard deviation, * = item with multiple answer option

Table 3 shows the items representing parents’ experience with MC, their children’s symptoms, reasons to seek medical advice, and the preferred treatment. The majority of parents (55,2%) had children previously having MC; about one-third had children currently having MC. Most of their children (79%) suffered from moderate discomfort (mean VAS score 6.1 on a scale of 0–10), itch being the most frequently reported. Of parents who had experience with MC (N = 81), the majority visited their GP (60.5%), primarily requesting treatment. However, advice to wait-and-see was given most often. When asked about the preferred treatment, most parents preferred an ointment to resolve MC, followed by cryotherapy. In contrast, only 6.7% said they would prefer to wait and see.

**Table 3:** Parent experience and treatment preferences

**Parents having children with current MC (N = 105)**	
Yes (%)	36 (34.3)
No (%)	69 (65.7)
**Parents having children with previous MC (N = 105)**	
Any previous (%)	58 (55.2)
Never (%)	47 (44.8)
**Presence of symptoms in any current/previous MC (N = 81)**	
No symptoms (%)	17 (21.0)
Symptoms (%)	64 (79.0)
Type of symptoms* (N = 64)	
Itch (%)	48 (75.0)
Inflammation (%)	33 (51.6)
Child concerned cosmetically (%)	34 (53.1)
Pain (%)	31 (48.4)
Parent concerned cosmetically (%)	23 (35.9)
Other (%)	10 (15.6)
**Discomfort (VAS score) of symptoms (N = 64)**	
Mean VAS score (0–10) (SD)	6.1 (2.1)
**Visited GP for MC (N = 81)**	
No (%)	32 (39.5)
Yes (%)	49 (60.5)
Reason for visit (if visited)* (N = 49)	
Treatment (%)	36 (73.5)
Information (%)	23 (46.9)
Other (%)	3 (6.1)
Advice or treatment received* (N = 49)	
Wait and see (%)	33 (67.3)
Ointment for itch or pain (%)	13 (26.5)
Ointment to resolve MC (%)	9 (18.4)
Cryotherapy (%)	7 (14.3)
Curettage (%)	1 (2.0)
Puncture (%)	0 (0)
Other (%)	3 (6.1)
Reason for visit (if not yet visited)* (N = 32)	
Treatment (%)	18 (56.3)
Information (%)	5 (15.6)
Possible spread (%)	4 (12.5)
Other (%)	9 (28.1)
Preferred treatment if symptomatic MC* (N = 105)	
Ointment to resolve MC (%)	50 (47.6)
Cryotherapy (%)	46 (43.8)
Ointment for itch or pain (%)	36 (34.3)
Curettage (%)	30 (28.6)
Expectative (%)	7 (6.7)
Other (%)	2 (1.9)

MC = molluscum contagiosum, N = number of responding parents, GP = general practitioner, * = item with multiple answer option, SD = standard deviation

### Willingness to participate in proposed interventional research

Only 27 GPs (20.9%) were willing to participate; most were either not, or in doubt. The reason most frequently mentioned was not supporting curettage or cryotherapy as a treatment. Other reasons most frequently mentioned (in open-text fields) were primarily practical in nature, e.g. not having the time or means, or not owning the clinic. The GPs estimated that on average 30.2% of parents would be willing to let their child(ren) participate (Table 4).

**Table 4:** Potential trial participation

**GPs willingness to participate**	
**Willing to participate in Mollusca trial (N = 129)**	
Yes (%)	27 (20.9)
No (%)	53 (41.1)
Doubt (%)	49 (38.0)
Reason for unwillingness or doubt* (N = 102)	
Non-supportive of curettage (%)	33 (32.4)
Non-supportive of cryotherapy (%)	21 (20.6)
Not willing to perform curettage (%)	19 (18.6)
Not meaningful (%)	15 (14.7)
Not feasible (%)	6 (5.9)
Not willing to perform cryotherapy (%)	6 (5.9)
Other (mostly practical in nature) (%)	43 (42.2)
**According to GP estimated % of parents willing to participate**	
Mean % (SD)	30.2 (21.8)
**Parents willingness to let their children participate**	
**Willing to participate in trial in case of MC (N = 105)**	
Yes (%)	61 (58.1)
No (%)	20 (19.0)
In doubt (%)	24 (22.9)
**Reason for unwillingness or doubt (N = 44)**	
Potential side effects (pain, scar formation) (%)	19 (43.2)
Expecting little or no symptoms (%)	12 (27.3)
Rather choose treatment (no randomization) (%)	11 (25.0)
Opting out for curettage (%)	10 (22.7)
Opting out for cryotherapy (%)	4 (9.1)
Opting out expectative management (%)	0 (0)
Other (%)	8 (18.2)
**Willing to participate if able to choose Tx (N = 105)**	
Yes (%)	80 (76.2)
No (%)	25 (23.8)
**If participating, objection if treatment not provided by own GP (N = 105)**	
No objection (%)	85 (81.0)
Yes, objection (%)	13 (12.4)
Doubt (%)	7 (6.7)

GP = general practitioner, N = number of responding GPs OR parents, * = item with multiple answer option, SD = standard deviation

In contrast, the majority of parents (58.1%) were willing to let their children participate. Those not willing or in doubt, most frequently mentioned potential side effects as the primary concern, followed by expecting little or no symptoms from MC, preferring to be able to choose treatment, or rather not having curettage. If choosing the treatment would be an option, more parents (76.2%) would be willing to let their child enroll, an increase of 18.1%. The majority of parents would not object if the treatment would be performed by research staff and not their own GP.

### Factors influencing participation willingness

The GPs’ baseline characteristics and their preferences were used in univariate logistical regression analysis to calculate the odds of a GPs willingness to participate. Age, estimating ≥ 50% of parents would request treatment, having experience with, or currently applying curettage, all significantly increased the odds of a GP being willing to participate in the proposed trial (Appendix 3, Table S1).

Regarding parents, only those choosing cryotherapy or curettage as a preferred treatment option, had significantly higher odds of letting their children participate (Appendix 3, Table S2).

## Discussion

### Summary

This survey demonstrated a significant gap between parents’ expectations and the GPs’ preferred approach in case of MC. The preferred approach for the majority of GPs was expectative, reflecting the current guidelines. In contrast, the majority of parents did, or would visit their GP, primarily requesting treatment to get rid of the lesions. This could be explained by the presence of symptoms and level of discomfort reported. However, if treatment was considered, symptom relief would be the primary aim; cryotherapy or curettage were rarely applied and only a minority of GPs were experienced with applying these treatments for MC.

Regarding the potential for trial participation, most GPs were not willing because of not supporting or unwilling to perform curettage or cryotherapy for MC. In addition, a lack of time or means, and other practical reasons were frequently mentioned. In contrast, the majority of parents were willing to participate; potential side effects or expecting only little symptoms caused by MC were most frequently mentioned by those unwilling or in doubt to participate.

### Strengths and limitations

Although not the first time that patient perspectives on MC are investigated [24], to our knowledge this is the first time a survey directly inquires about the treatment preferences and expectations of both GPs and parents, providing valuable insight into the current approach to MC.

The discrepancy between parents expectations and the preferred approach of GPs found in this survey, forms an argument to continue efforts finding effective and acceptable treatments for MC. Regarding the feasibility of a potential interventional study as proposed, this survey points out that finding GPs willing to participate when requesting to perform cryotherapy or curettage, would be a major challenge.

A limitation of this study is that both surveys were unique, i.e. not standardized or validated. However, all questions were thoroughly reviewed by the authors and the department’s research committee. Nonetheless, common errors inherent to newly constructed surveys cannot be ruled out.

As both surveys were distributed online and response was optional, generalizability of the results may be limited due to potential bias caused by selective (non-)response from GPs and parents.

Only completed surveys were analyzed. Due to the anonymity, we could not determine the reasons for not completing a survey. However, we have no reason to believe respondents either completing the survey or not, would fundamentally be different. The registration of dates and times of the surveys actually suggest that incomplete surveys possibly originate from the same respondents, re-opening a new survey, for example for technical reasons.

Finally, this survey was specifically designed for Dutch parents and GPs, in the Dutch primary healthcare setting. Therefore our results might not be generalizable to other primary healthcare settings; cultural aspects influence the views and opinions of respondents in other countries. For example, patients in the US or the UK may even have a stronger wish for intervention and expect to be treated by their physician or GP.

### Comparison with existing literature

Browning et al. recently reported patient perceived outcome from a large trial evaluating berdazimer gel, primarily focusing on patient perceived clearance of MC lesions [24]. The 891 trial participants all rated improvement i.e. clearance. However, 30 participants also received an in-depth exit interview, showing a significant impact on psychosocial wellbeing and confirming the same desire from parents for MC to be treated. Our survey differed in that it included responding parents from the general public instead of only trial participants and included the perspective of GPs, providing a more comprehensive view on the expectations and preferences of both groups.

## Conclusions

This study demonstrates that GPs primarily prefer an expectative approach to MC, illustrating adherence to current guidelines. However, parents report significant symptoms and burden and prefer their children to be treated. This discrepancy underlines the importance of continuing the search for effective treatments for MC; clinical trials would be well suited for this purpose. However, as far as clinical trials in primary care are concerned, finding sufficient GPs willing to contribute seems a concern. Investigating more widely accepted treatments seems less meaningful since most of these options have not been successful so far [17]. If we want to adhere to the investigation of potentially more effective destructive treatments, selecting a group of GPs willing to perform these interventions would likely be necessary.

### Electronic supplementary material

Below is the link to the electronic supplementary material.

Supplementary Material 1

## Data Availability

The datasets used and analysed during the current study are available from the corresponding author on reasonable request.

## Footnotes

1 *WMO stands for ‘Wet Medisch-wetenschappelijk Onderzoek met mensen’, meaning ‘Medical Research Involving Human Subjects act’ in English.

## Abbreviations

EDC: Electronic Data Capture
ELAN: Extramural LUMC Academic Network
GP: General practitioner
IQR: Interquartile range
LUMC: Leiden University Medical Center
MC: Molluscum contagiosum
MCV: Molluscum contagiosum virus
NICE: National Institute for Health and Care Excellence
UK: United Kingdom
