# Supplementary material for: Molluscum contagiosum survey – common approach and attitude towards treatment and research in Dutch general practice
Source: BMC Prim Care. 2023 Dec 7;24:264. doi: 10.1186/s12875-023-02226-y (PMC10702083; doi:10.1186/s12875-023-02226-y)
Supplement: Supplementary file 1 — Supplementary Material 1 [file 12875_2023_2226_MOESM1_ESM.docx]

## Additional files

### Appendix 1. Mollusca survey – questionnaire for general practitioners


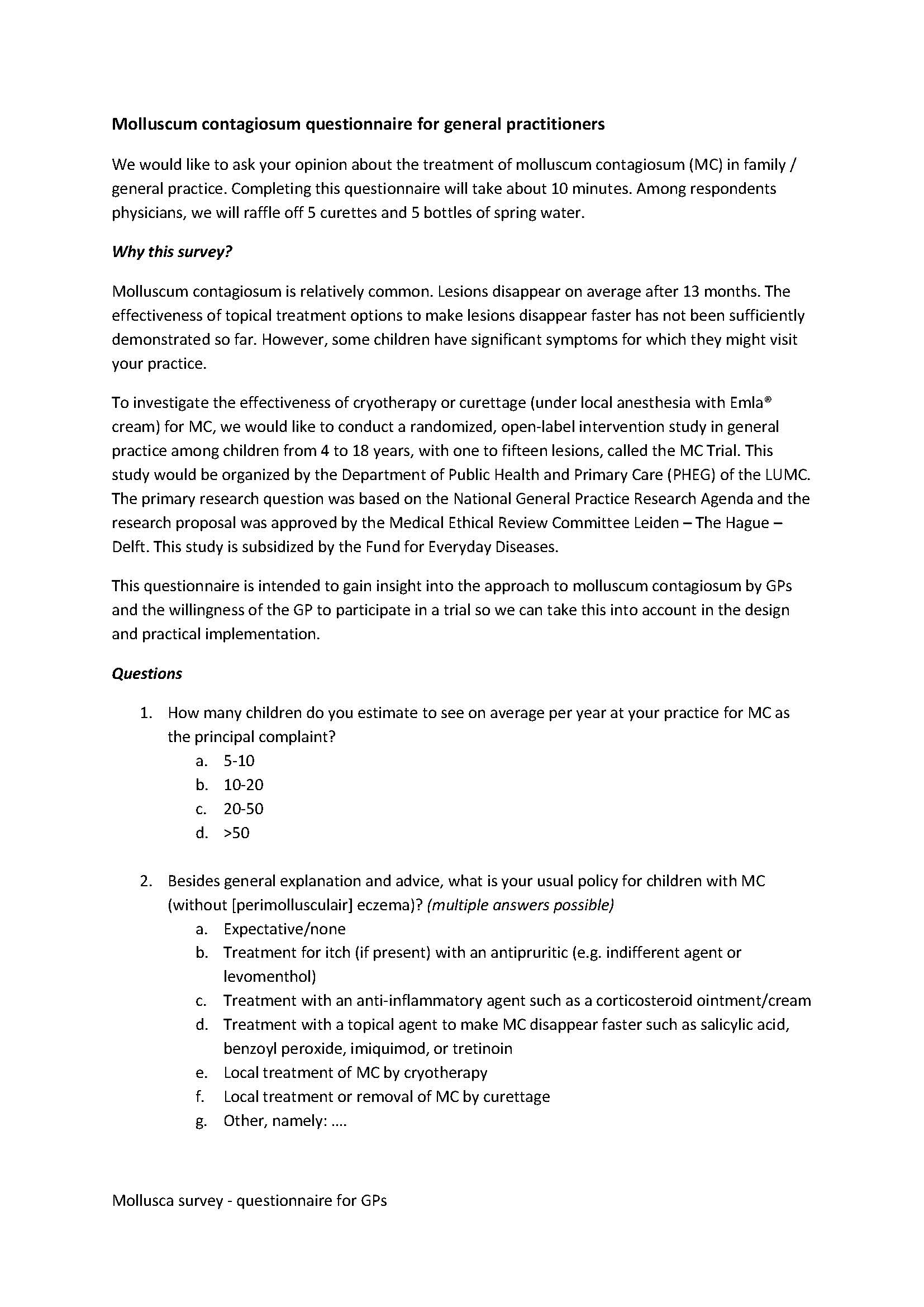


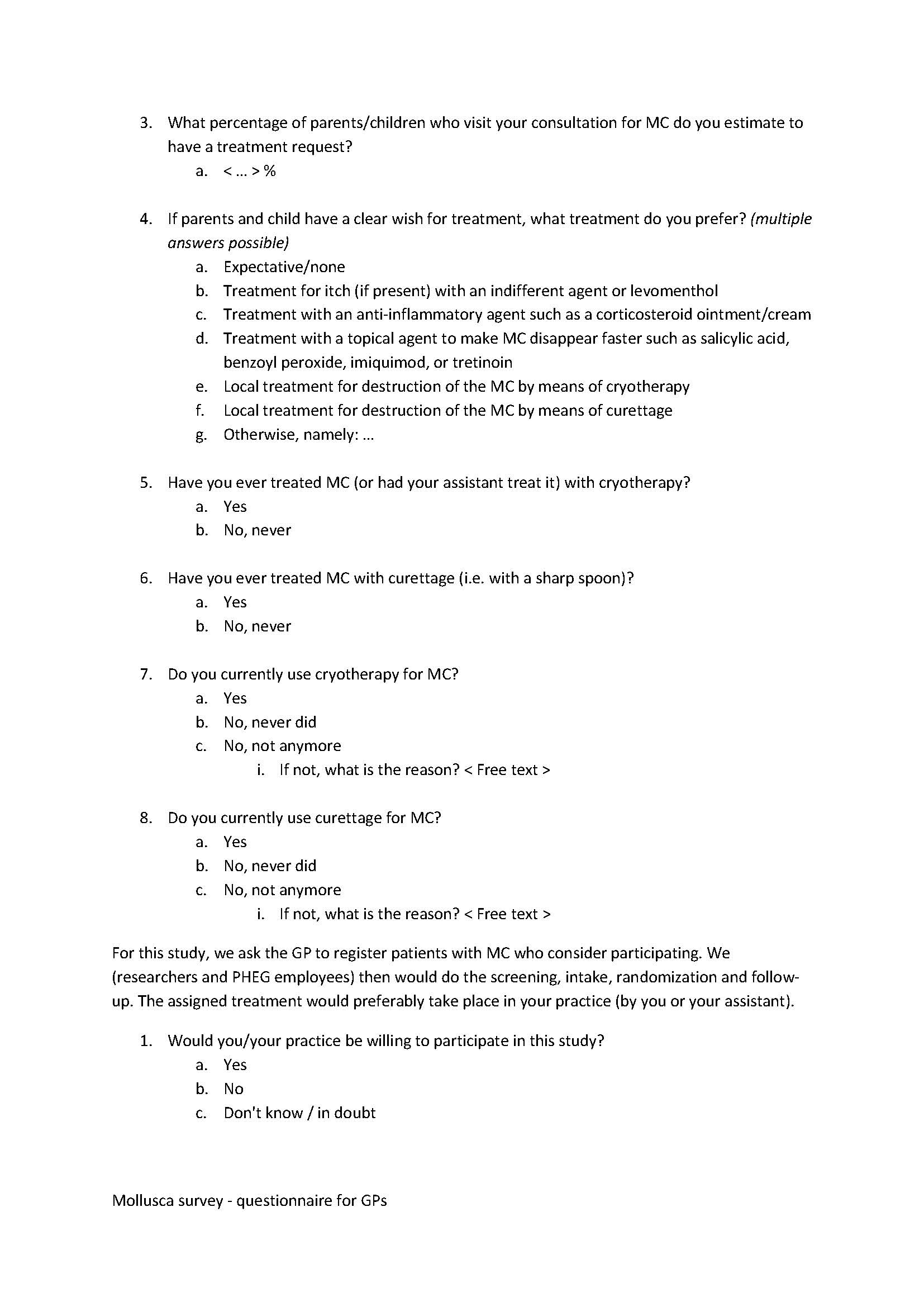


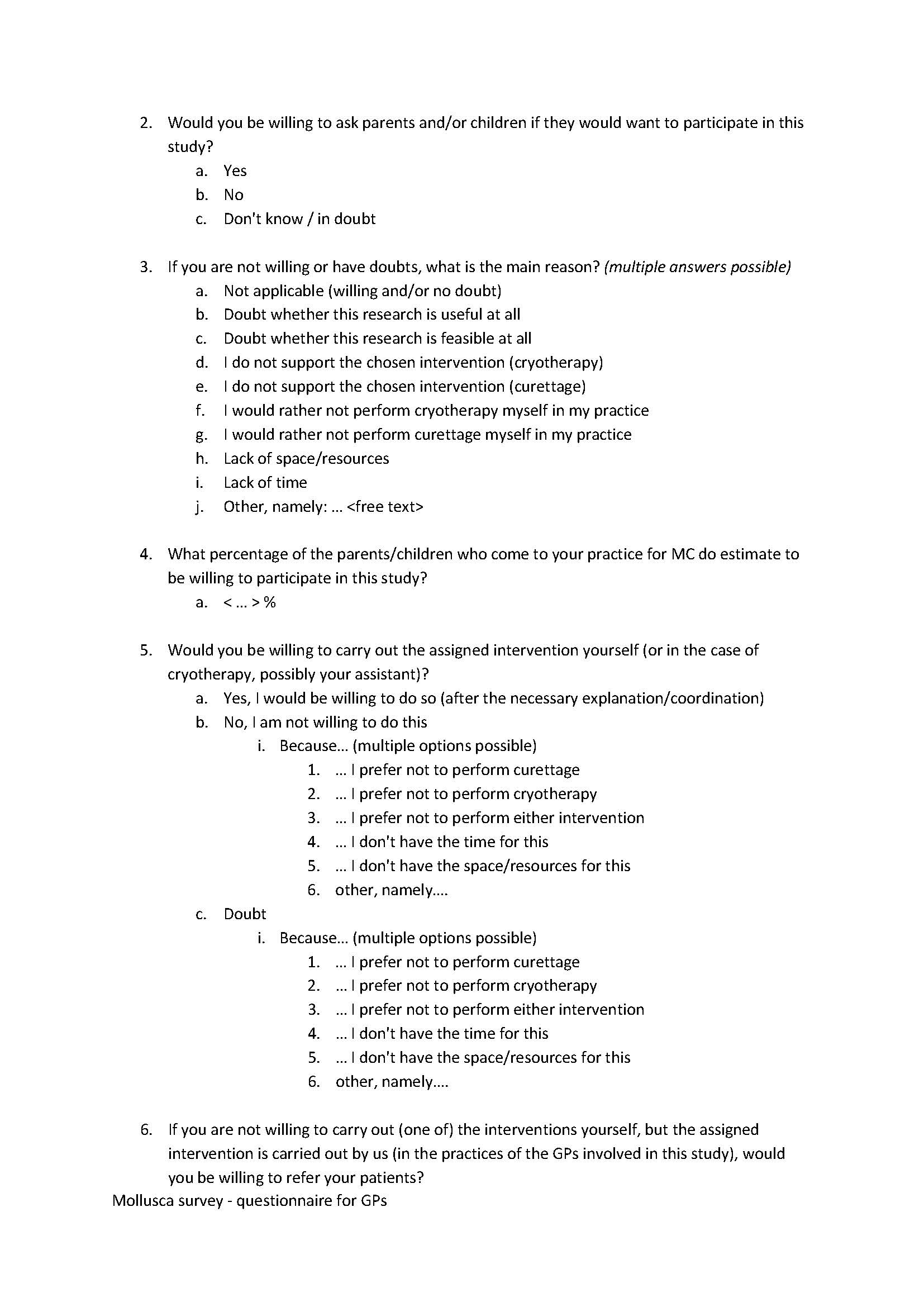


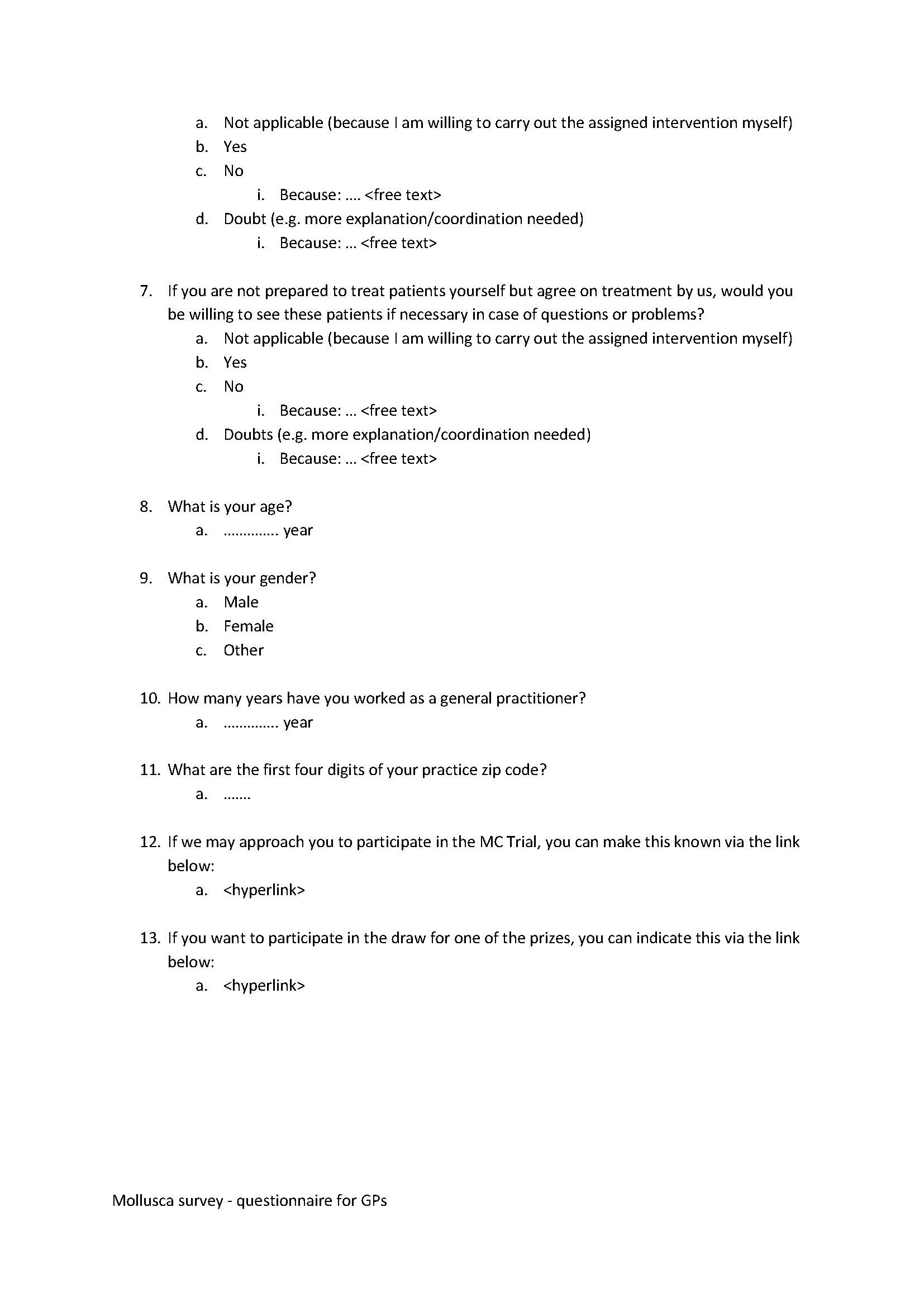


### Appendix 2. Mollusca survey – questionnaire for parents / caregivers


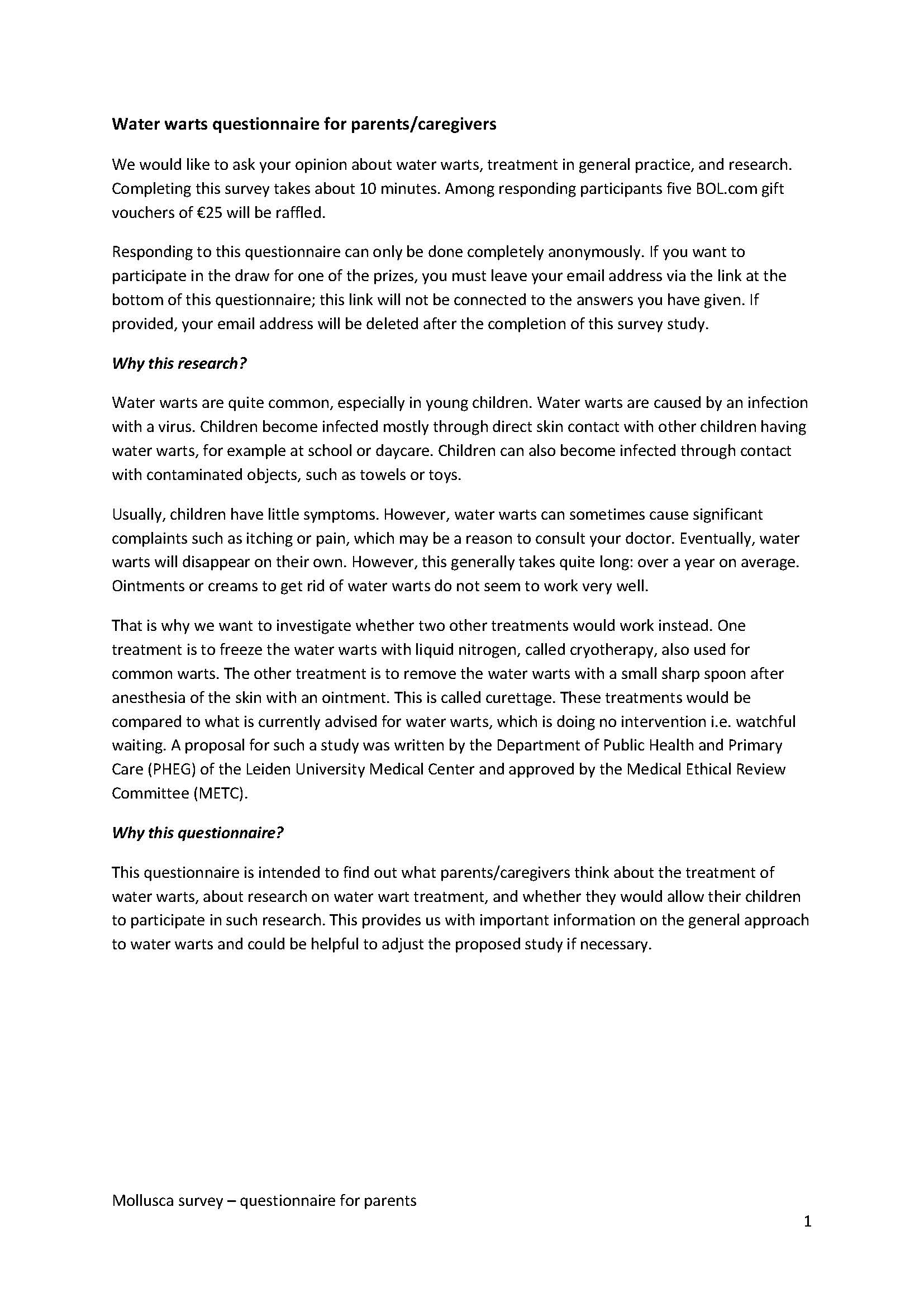

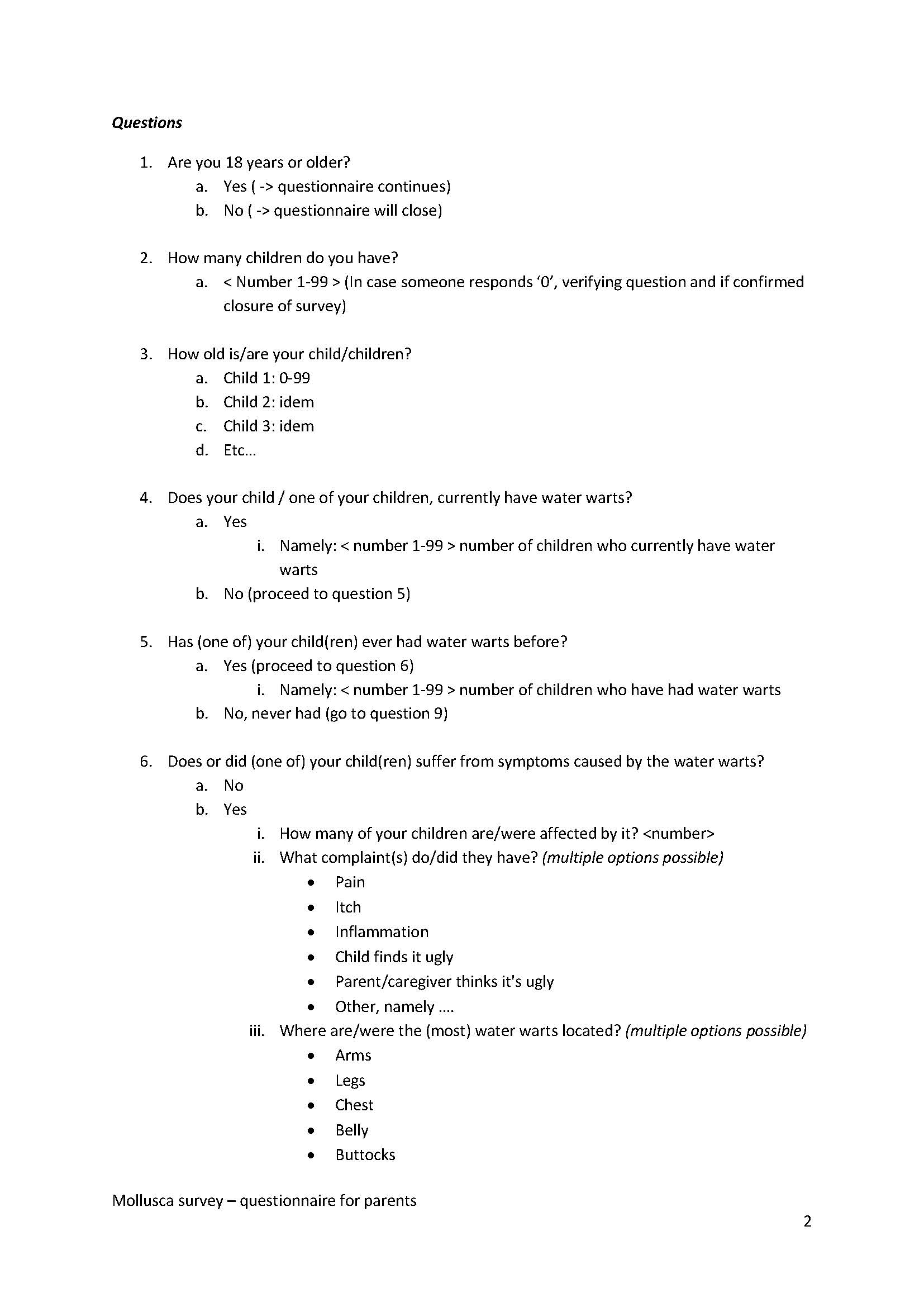

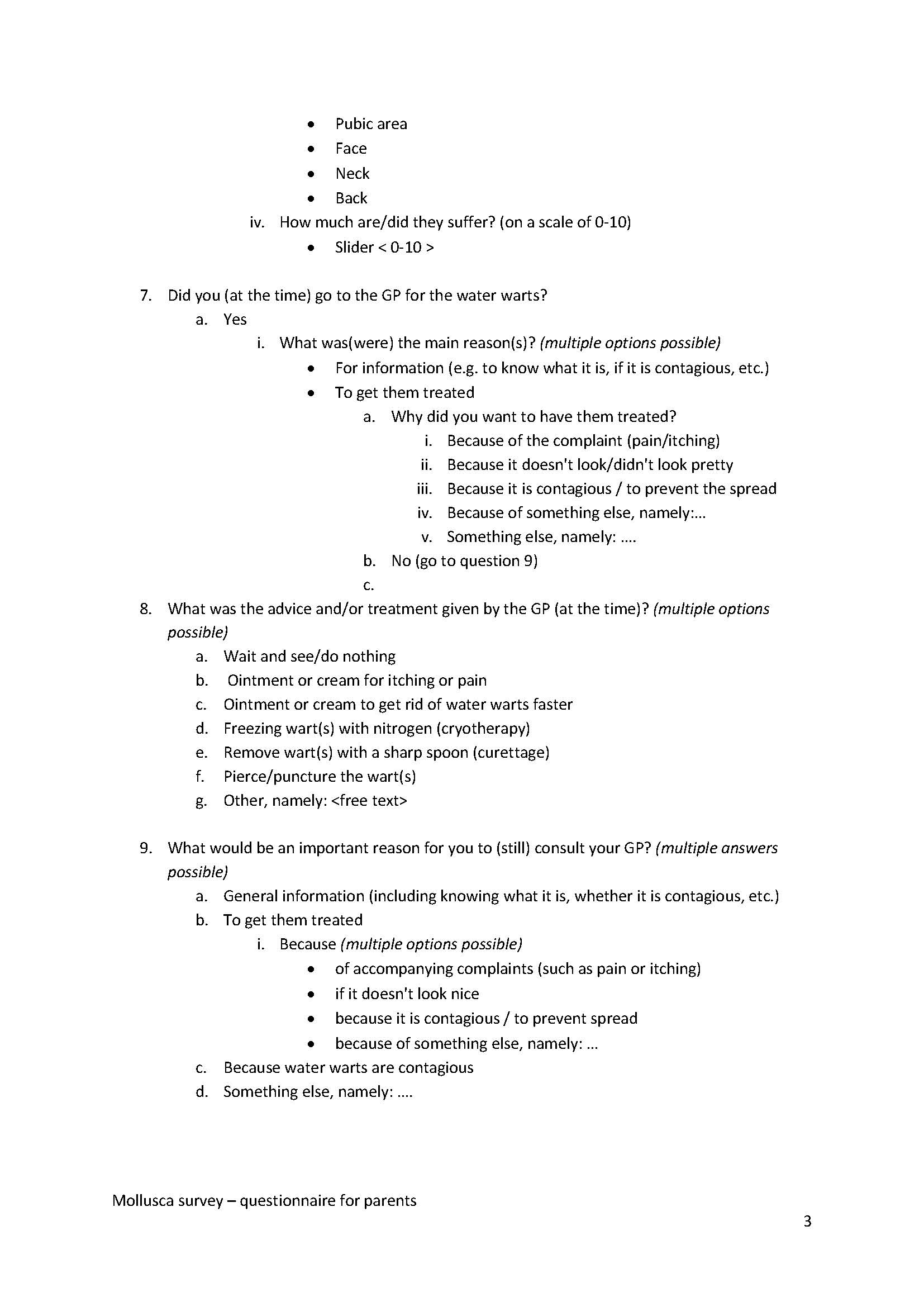

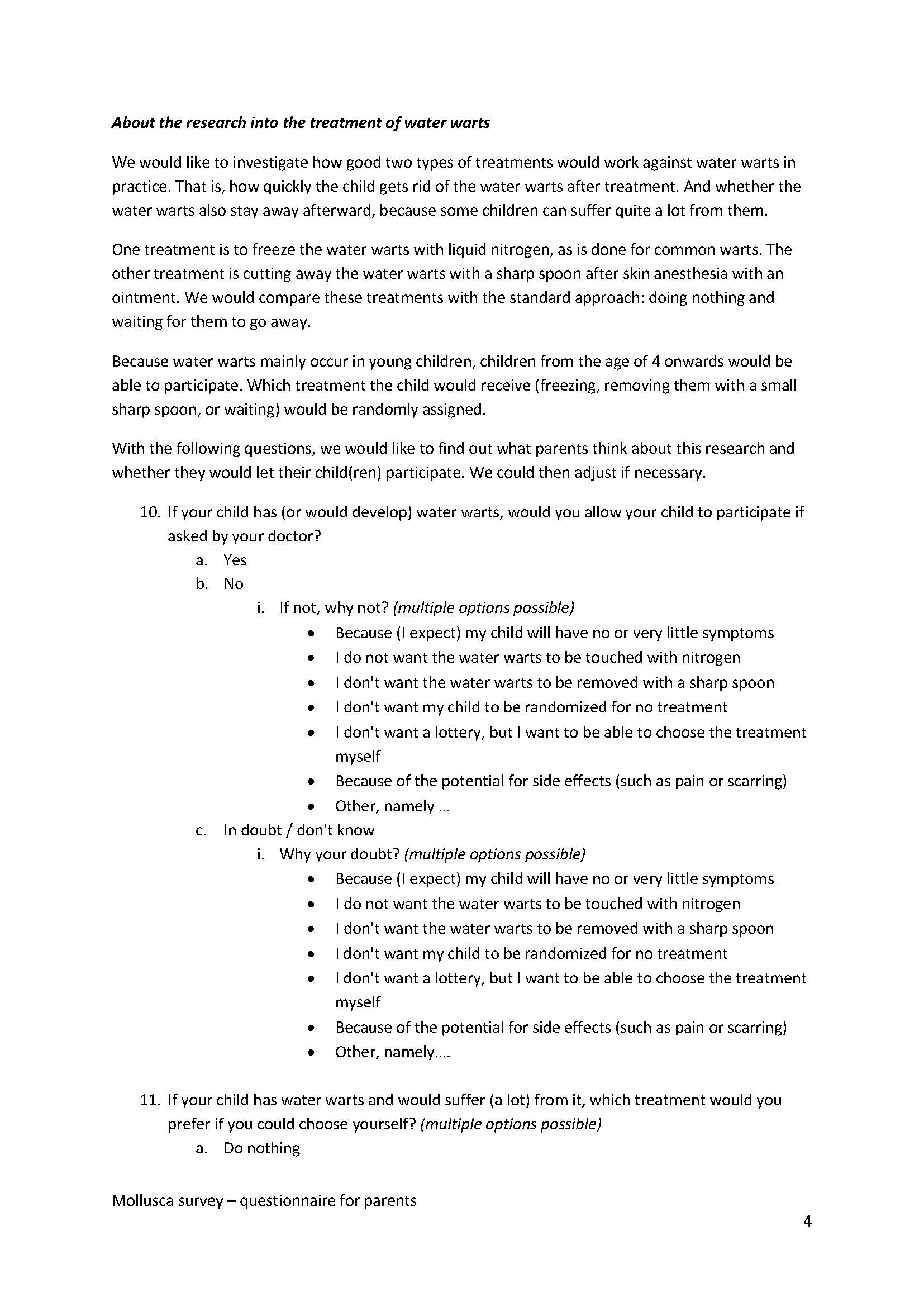

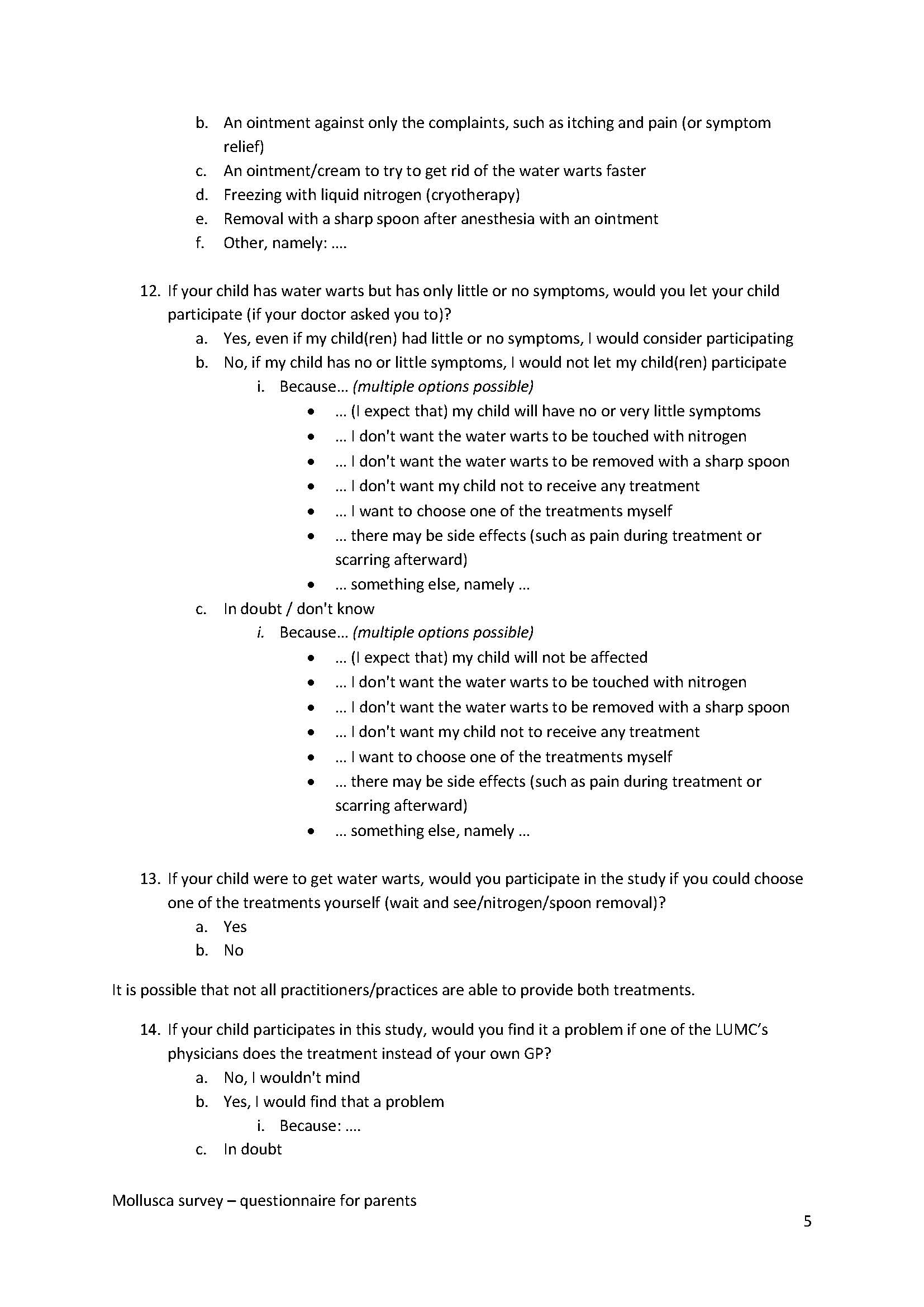

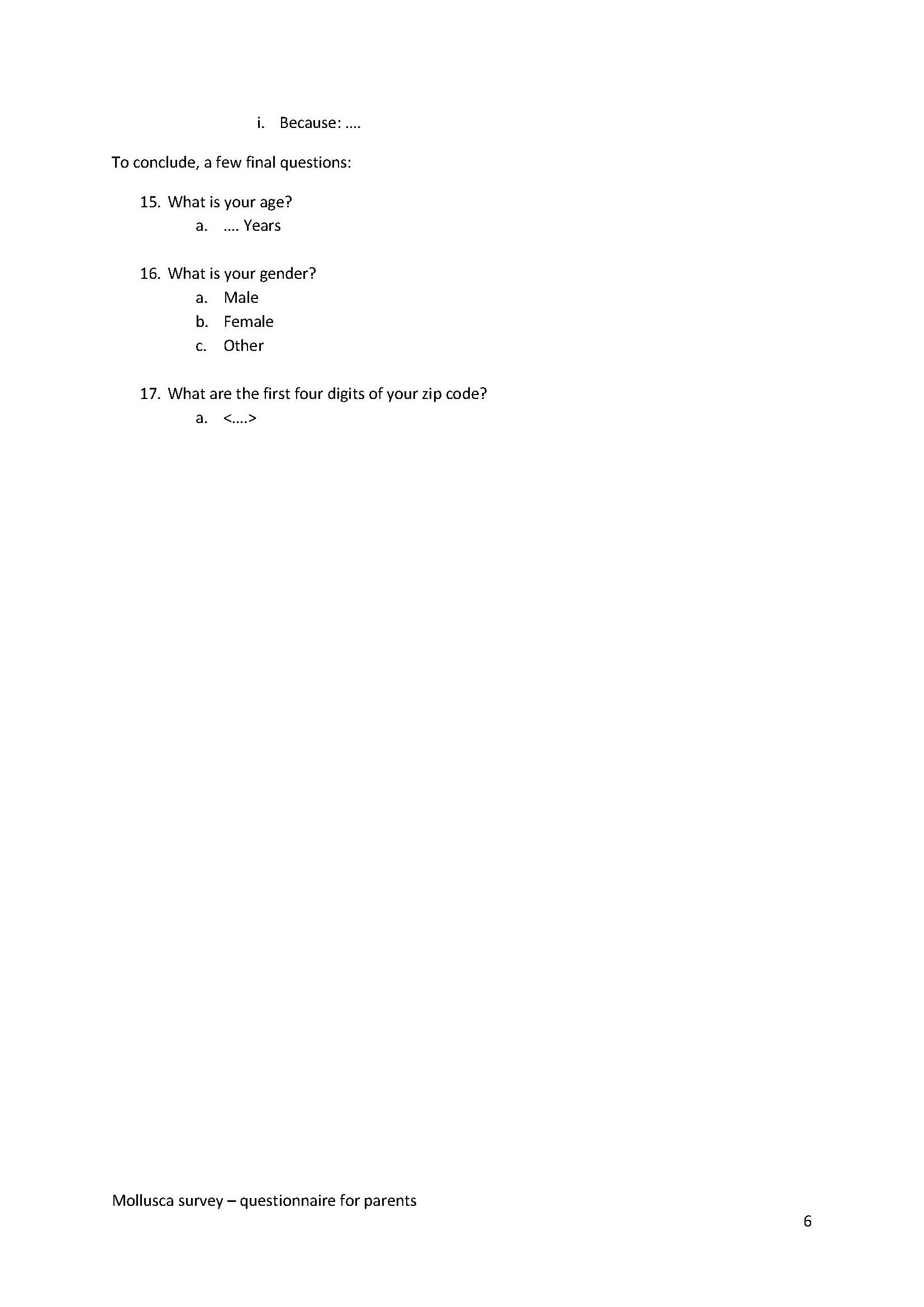


### Appendix 3. Logistical regression for participation in proposed study

| Table S1. Logistical regression for GPs’ willingness to participate | | | |
| --- | --- | --- | --- |
|  | *Univariate analysis* | | |
|  | OR | 95% CI | p-value |
| Baseline characteristics |  |  |  |
| Age (years) | **1.046** | 1.002-1.092 | **0.040** |
| Gender (female) | 1.029 | 0.391-2.704 | 0.954 |
| Working experience >10 years | 1.709 | 0.702-4.161 | 0.238 |
| Experience and preferences |  |  |  |
| Est. MC visits ≥10 yearly | 0.925 | 0.353-2.426 | 0.874 |
| Est. parents requesting Tx ≥50% | **3.147** | 1.315-7.530 | **0.010** |
| Expectative Tx preferred in general (yes) | 1.169 | 0.308-4.435 | 0.819 |
| Expectative Tx preferred if Tx request (yes) | 0.599 | 0.255-1.406 | 0.239 |
| Experience with cryotherapy for MC (yes) | 1.920 | 0.804-4.585 | 0.142 |
| Experience with curettage for MC (yes) | **3.697** | 1.411-9.690 | **0.008** |
| Currently applying cryotherapy for MC (yes) | 1.633 | 0.601-4.440 | 0.336 |
| Currently applying curettage for MC (yes) | **5.600** | 1.700-18.446 | **0.005** |

*OR = odds ratio, 95% CI = 95% confidence interval, MC = molluscum contagiosum, Est. = estimated, Tx = treatment (statistically significant in purple)*

| Table S2. Logistical regression for parents’ willingness to participate | | | |
| --- | --- | --- | --- |
|  | *Univariate analysis* | | |
|  | OR | 95% CI | p-value |
| Baseline characteristics |  |  |  |
| Age (years) | 1.008 | 0.938-1.084 | 0.824 |
| Gender (female) | 0.578 | 0.166-2.012 | 0.389 |
| Number of children (≥2) | 0.931 | 0.566-1.532 | 0.779 |
| Age of children, mean (years) | 0.975 | 0.880-1.081 | 0.975 |
| Experience and preferences |  |  |  |
| Children w current MC (yes) | 1.209 | 0.531-2.750 | 0.651 |
| Children w previous MC (yes) | 0.651 | 0.296-1.430 | 0.285 |
| Symptoms in case of MC (yes) | 2.229 | 0.750-6.619 | 0.149 |
| VAS score in case of MC (0-10) | 1.125 | 0.972-1.301 | 0.113 |
| Visited GP for MC (yes) | 1.952 | 0.789-4.826 | 0.148 |
| Preferred Tx cryotherapy/curettage (yes) | **3.794** | 1.672-8.607 | **0.001** |

*OR = odds ratio, 95% CI = 95% confidence interval, MC = molluscum contagiosum Tx = treatment (statistically significant in red)*
